# Which is the real nature of glucose‐dependent insulinotropic peptide?: Endogenous vs pharmacological

**DOI:** 10.1111/jdi.14357

**Published:** 2024-11-14

**Authors:** Yuji Yamazaki, Yutaka Seino

**Affiliations:** ^1^ Center for Diabetes, Endocrinology and Metabolism Kansai Electric Power Hospital Osaka Japan; ^2^ Yutaka Seino Distinguished Center for Diabetes Research Kansai Electric Power Medical Research Institute Kobe Japan

## Abstract

GIP is a multifaceted hormone whose role in metabolism is highly context‐dependent. Pharmacological GIP receptor activation promotes weight loss and improves insulin sensitivity, contrasting sharply with the lipogenic and insulin‐resistant effects of endogenous GIP. However, it remains unclear whether these effects simply amplify endogenous GIP's actions or represent distinct mechanisms.

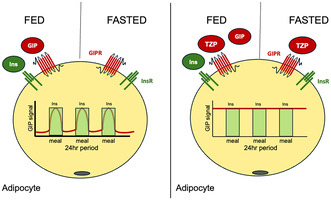

Glucose‐dependent insulinotropic peptide (GIP) is an incretin hormone playing a crucial role in glucose regulation, secreted by K‐cells in the small intestine in response to nutrient intake. GIP enhances insulin secretion in a glucose‐dependent manner. However, its insulinotropic effects are short‐lived due to rapid degradation by dipeptidyl peptidase‐4 (DPP‐4), which is consistent with a basic model of incretins in which an enteric hormone controls insulin secretion of pancreatic beta cells minimizing the fluctuation of postprandial glycemia. Besides their pancreatic action, incretins have various extrapancreatic actions that are also relevant to feeding and nutrient metabolism.

Incretin has been recognized as a relevant drug target for diabetes treatment, because of its glucose‐dependent insulinotropic effects. However, GIP was not successfully used for diabetes treatment, whereas Glucagon‐like peptide 1 (GLP‐1), the other incretin that was later characterized, was in line with a drug for diabetes, so‐called a GLP‐1 receptor agonist. In addition to its excellent hypoglycemic effect, it also has a weight loss effect, which is a preferred factor widely used throughout the world. The additional benefits such as renal function and CVOTs (cardiovascular outcome trials) have been verified, which have also increased its usefulness.

Recently developed GIP/GLP‐1 dual receptor agonists, such as tirzepatide, have demonstrated surprising results. Clinical trials have shown that these dual agonists not only improve glycemic management but also lead to significant weight reductions. Tirzepatide is a synthetic compound designed by modifying GIP, giving it a dual action as an agonist for both GIP and GLP‐1 receptors. It binds to the GIP receptor with an affinity comparable to that of endogenous GIP, meaning it effectively activates the GIP pathway similarly. However, its affinity for the GLP‐1 receptor is approximately 10 times lower than that of endogenous GLP‐1, which results in a differential activation of the two receptors. The chimeric composition of its peptide sequence may act as a biased agonist, selectively activating certain GIP‐mediated pathways. While the full mechanism of tirzepatide remains unclear, GIP's potential in therapeutic applications has been revisited with new findings on GIP. Moreover, key questions have been raised about whether the pharmacological effect of tirzepatide can be explained with just an enhancement of intrinsic GIP function.

The early report demonstrated that GIP receptor knockout (GIPR−/−) mice is resistant to diet‐induced obesity^1^. In particular, GIP has been shown to promote glucose uptakes and TG accumulation into adipocyte cell lines in the presence of insulin^1^, ^2^. Several papers were published in the same direction, which raised concerns about its contribution to obesity and insulin resistance, particularly in conditions of overnutrition. A genome‐wide association study (GWAS) has identified that variations in genes related to the GIP signaling pathway are associated with obesity^3^. Specifically, GIPR (GIP receptor) gene variants have been linked to higher body mass index (BMI) and increased fat accumulation. These findings suggest that altered GIP signaling may contribute to metabolic disorders like obesity by influencing how the body processes nutrients and stores fat, suggesting that GIP might act as a “thrifty gene.” This concept refers to genes that enhance fat storage and energy conservation, which may have provided an evolutionary advantage during times of food scarcity. However, in modern environments with constant food availability, GIP's lipogenic effects in adipose tissue may exacerbate obesity, fueling a cycle of insulin resistance and hyperglycemia. This positions GIP as a key factor in energy regulation and metabolism.

Despite the limitations of endogenous GIP in type 2 diabetes, its therapeutic potential has garnered interest. The advent of GIP/GLP‐1 dual receptor agonist holds promise for the therapeutic potential of GIP. The GIP/GLP‐1 dual receptor agonist, tirzepatide, binds to both GIP and GLP‐1 receptors, leading to hypoglycemic action, and significant weight loss. Several hypothetical models have come up. The combination of both GIP and GLP‐1 receptor activations might be essential to exhibit this drug effect, Tirzepatide might work on the GIP receptor antagonistically, and Tirzepatide acts as a superagonist at the GLP‐1 receptor. However, recent findings raised the idea that pharmacological GIP receptor activation offers benefits distinct from those of endogenous GIP. GIP receptors are expressed in key areas of the brain that control hunger and satiety, such as the arcuate nucleus. Activation of these receptors can influence food intake and energy expenditure by modulating neural circuits involved in appetite control as well as recognition of the central effects on appetite regulation by GLP‐1. Han *et al*.^4^ demonstrated that GIP plays a crucial role in appetite regulation via its actions in the central nervous system, specifically in the hypothalamus. Their findings suggest that the pharmacological level of GIP receptor activation in the brain could influence neural pathways responsible for energy balance, potentially enhancing satiety and reducing food intake (Figure 1). This mechanism, combined with tirzepatide's dual receptor activation, may help explain its pronounced effects on appetite suppression and fat mass reduction.

**Figure 1:**
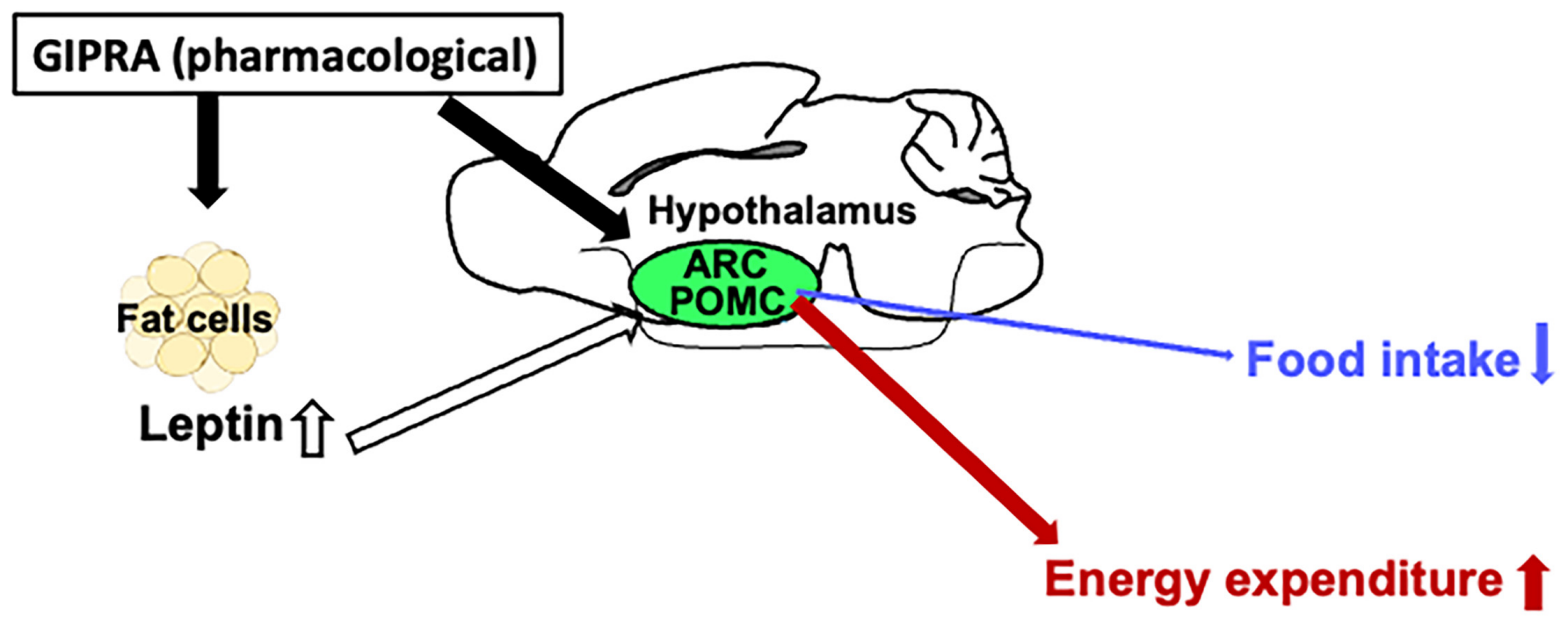
Schematic model of the pharmacological glucose‐dependent insulinotropic peptide (GIP) action in the brain. Pro‐opiomelanocortin (POMC)‐expressing neurons in the arcuate nucleus (ARC) of the hypothalamus are activated in a long‐acting GIP receptor agnostic‐treated DIO mice, leading to the acute decrease of food intake, and the increase of energy expenditure. The mechanisms might involve rises in leptin derived from adipocytes stimulated by GIP. GIPRA, GIP receptor agonist. The schematic model referred to Han *et al*.^4^

Moreover, pharmacological GIP overcomes the impaired β‐cell response seen with endogenous GIP in people with diabetes, suggesting that it can exert beneficial metabolic effects when administered pharmacologically. A striking effect of pharmacological GIP receptor activation is weight loss, particularly when combined with GLP‐1 receptor activation. Endogenously, GIP promotes fat storage, but pharmacological activation appears to reverse this. The opposite results suggest that pharmacological activation of GIPs may be involved in energy expenditure for fat burning. Recently, Regmi *et al*.^5^ hypothesized that tirzepatide modulates adipocyte metabolism by continuously activating the GIP receptor, even in the absence of insulin. This sustained GIP activation is proposed to enhance fat efflux from adipocytes, thereby increasing fat oxidation and improving overall energy balance (Figure 2). The hypothesis suggests that tirzepatide's dual GIP and GLP‐1 agonism creates a potent metabolic effect that could be leveraged to treat obesity by promoting fat loss and improving insulin sensitivity.

**Figure 2:**
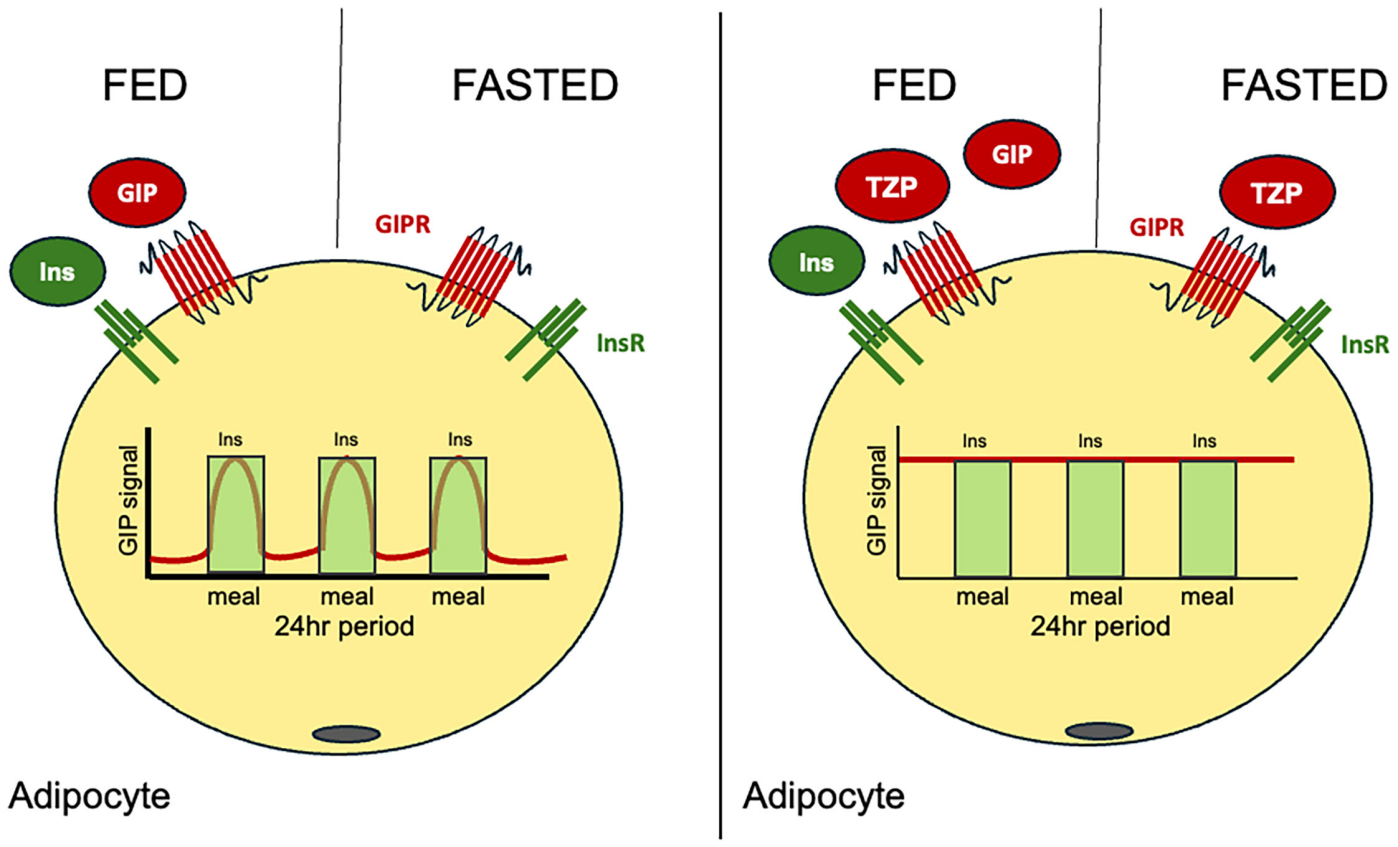
Hypothetical mechanism of the pharmacological Glucose‐dependent insulinotropic peptide (GIP) action in the adipocyte. Only at the postprandial state, endogenous GIP directly enhance nutrient partitioning into adipocytes in the presence of insulin. On the contrary, in the case of tirzepatide treated, tirzepatide constitutively activates GIP signaling, and particularly, the pharmacological GIP receptor agonism enhances lipolysis in the fasting state, enhancing the nutrient efflux from adipocytes. This schematic model referred to Regmi *et al*.^5^ GIP signal indicated by red line, Insulin signal indicated by light green squares, GIPR, GIP receptor; Ins, insulin; InsR, insulin receptor; TZP, tirzepatide.

GIP is a multifaceted hormone whose role in metabolism is highly context‐dependent. The differences between endogenous and pharmacological GIP highlight the complexity of its role in metabolism. Endogenously, GIP plays an essential role in glucose regulation, lipid metabolism, and β‐cell function. However, its lipogenic and insulin‐resistant effects in conditions like obesity and type 2 diabetes complicate its role. Pharmacologically, GIP receptor agonists offer a promising approach by harnessing the hormone's beneficial aspects while minimizing its drawbacks. Pharmacological GIP receptor activation promotes weight loss and improves insulin sensitivity, contrasting sharply with the lipogenic and insulin‐resistant effects of endogenous GIP. While pharmacological concentrations of GIP, such as those achieved with tirzepatide, demonstrate significant metabolic benefits, it remains unclear whether these effects simply amplify endogenous GIP's actions or represent distinct mechanisms. Further research is necessary to fully understand the mechanisms driving these differences and to unveil the “true” nature of GIP and its therapeutic potential.

## DISCLOSURE

YY received honoraria for lectures from Sumitomo Pharma Co., Eli Lilly Japan K.K. and Mitsubishi Tanabe Pharma Corporation, and YS has received grants from Nippon Boehringer Ingelheim Co., Ltd., ARKRAY Marketing, Inc., Taisho Pharmaceutical Co., Ltd., Novo Nordisk Pharma Ltd., Terumo Corporation and Sumitomo Pharma Co.; and honoraria for lectures from Taisho Pharmaceutical Co., Ltd., Nippon Becton Dickinson Company, Ltd., Novo Nordisk Pharma Ltd., Eli Lilly Japan K.K., Mitsubishi Tanabe Pharma Corporation, Sumitomo Pharma Co., Ltd. and Ono Pharmaceutical Co., Ltd. Yutaka Seino is an Editorial Board member of JOURNAL OF DIABETES INVESTIGATION and a co‐author of this article. To minimize bias, they were excluded from all editorial decision‐making related to the acceptance of this article for publication.

## FUNDING

There is no external funding/fellowship support.
